# Hepatocellular Carcinoma Immunotherapy and the Potential Influence of Gut Microbiome

**DOI:** 10.3390/ijms22157800

**Published:** 2021-07-21

**Authors:** Sally Temraz, Farah Nassar, Firas Kreidieh, Deborah Mukherji, Ali Shamseddine, Rihab Nasr

**Affiliations:** 1 Department of Internal Medicine, Hematology/Oncology Division, American University of Beirut Medical Center, Riad El Solh, Beirut 1107 2020, Lebanon; fn16@aub.edu.lb (F.N.); fk30@aub.edu.lb (F.K.); dm25@aub.edu.lb (D.M.); as04@aub.edu.lb (A.S.); 2 Department of Anatomy, Cell Biology and Physiology, American University of Beirut Medical Center, Riad El Solh, Beirut 1107 2020, Lebanon

**Keywords:** hepatocellular carcinoma, gut microbiome, microbiota, immunotherapy

## Abstract

Disruptions in the human gut microbiome have been associated with a cycle of hepatocyte injury and regeneration characteristic of chronic liver disease. Evidence suggests that the gut microbiota can promote the development of hepatocellular carcinoma through the persistence of this inflammation by inducing genetic and epigenetic changes leading to cancer. As the gut microbiome is known for its effect on host metabolism and immune response, it comes as no surprise that the gut microbiome may have a role in the response to therapeutic strategies such as immunotherapy and chemotherapy for liver cancer. Gut microbiota may influence the efficacy of immunotherapy by regulating the responses to immune checkpoint inhibitors in patients with hepatocellular carcinoma. Here, we review the mechanisms by which gut microbiota influences hepatic carcinogenesis, the immune checkpoint inhibitors currently being used to treat hepatocellular carcinoma, as well as summarize the current findings to support the potential critical role of gut microbiome in hepatocellular carcinoma (HCC) immunotherapy.

## 1. Introduction

Primary liver cancer comprises mainly hepatocellular carcinoma (HCC) in 75–85% of cases, followed by intrahepatic cholangiocarcinoma in 10–15%, in addition to other non-common types. In 2018, liver cancer accounted for 841,080 new cases and 781,631 new deaths worldwide, thereby rendering it the sixth most common cancer and the fourth most common cause of cancer-related death in the world; Ferlay, et al. [1]. Liver cancer is characterized by a poor prognosis with a 5-year survival of 18% unless discovered at an early stage where invasive treatment is the only resolution and includes ablation, surgical resection, or liver transplant. The majority of patients have advanced disease at diagnosis and, until recently, sorafenib was the only treatment option of systemic therapy for those patients. Currently, the standard of care in patients with advanced HCC involves immunotherapy combination of the checkpoint inhibitor atezolizumab and the targeted antibody bevacizumab.

The gut microbiota, also known as the ‘forgotten organ’, is the largest micro-ecosystem in the human body, which encompasses more than 1014 microorganisms. It is vital to the host’s metabolism and immune response, including antitumor response following immunotherapy and chemotherapy [2]. Although the liver is not in direct contact with the microbiota, it has a tight bidirectional link to the gut through the biliary tract, hepatic portal vein, and bile secretions [3,4]. However, dysbiosis, which is defined as qualitative and quantitative alterations of the gut microbiota, has the potential to destroy the gut barrier and increase intestinal penetrability. Moreover, the translocation of gut bacteria, bacterial overgrowth, and dysplasia of the immune system result in a condition known as “leaky gut” [5]. Both dysbiosis and the leaky gut are associated with a cycle of hepatocyte injury and regeneration characteristic of chronic liver disease, thereby encouraging the stepwise progression from fibrosis to cirrhosis and ultimately HCC.

In this review, we delve into the mechanism through which the gut microbiota impacts the pathogenesis of various liver diseases leading to HCC and summarize the current findings to support the potential critical role of gut microbiome in HCC immunotherapy.

## 2. Role of Gut Microbiota

The liver is supplied with blood from two sources: the hepatic artery, which originates from the celiac trunk, and the portal vein, which brings blood from the intestines and spleen. Blood carried through the portal vein is rich in nutrients and bacterial components like bacterial DNA, lipopolysaccharides (LPS), and peptidoglycan [6]. Kupffer cells, which are macrophages located in the sinusoids of the liver, eliminate these bacterial components under normal physiological conditions and prevent their harm to the body [7]. The gut microbiota also plays an important role in protecting the portal vein from invasion by pathogens through bacteriocins released by symbiotic bacteria that occupy the intestinal epithelium [8]. The gut microbiota also participates in gut immune maturation, such as the maturation of intestinal CD4^+^ and CD8^+^ T cells and dendritic cells [9].

Moreover, some metabolites that are produced by the gut microbiota regulate the physiological functions of the liver. The gut microbiota ferments dietary fibers to produce short-chain fatty acids, including butyric and propionic acid, which regulate proliferation and differentiation of liver cells and suppress inflammation in the liver by inducing regulatory T cells through an epigenetic mechanism [10]. The gut microbiota also breakdowns inulin, cellulose, and starch, which are termed indigestible carbohydrates, the end result of which is used by the hepatic cell for growth [11]. Another microbial metabolite resulting from polyphenolics, which are found in berries and pomegranates, is urolithin. This metabolite thwarts harmful substances from entering the portal vein [12]. Thus, gut homeostasis is essential for health.

## 3. Mechanisms by Which Gut Microbiota Induce HCC

There is a close link between dysbiosis and leaky gut; dysbiosis causes the intestinal barrier to be more permeable, whereas the leaky gut allows bacterial metabolites and microbiota-associated molecular patterns (MAMPs) to translocate and reach the liver.

Dysbiosis impacts metabolic pathways in the gut microbiota through production of bacterial metabolites such as bile acids. Gram-positive bacteria have an enhanced capacity for the conversion of bile acid to secondary bile acids [13]. Bacterially generated secondary bile acid deoxycholic acid (DCA) regulates liver sinusoidal cells (LSEC)- and CXCL16-dependent natural killer T cell (NKT) recruitment [14]. DCA was also found to increase levels of toll-like receptor 2 (TLR2) expression on hepatic stellate cells (HSCs), which in turn also increase TLR2 agonist lipoteichoic acid (LTA) in tumor promoting senescence-associated secretory phenotype (SASP) [15]. Moreover, DCA activates the mammalian target of rapamycin (mTOR) pathway in hepatocytes, ultimately resulting in HCC development [16]. In dysbiosis, short chain fatty acids specifically butyrate resulting from digestion of inulin have been found to promote HCC development [17].

**Figure 1:**
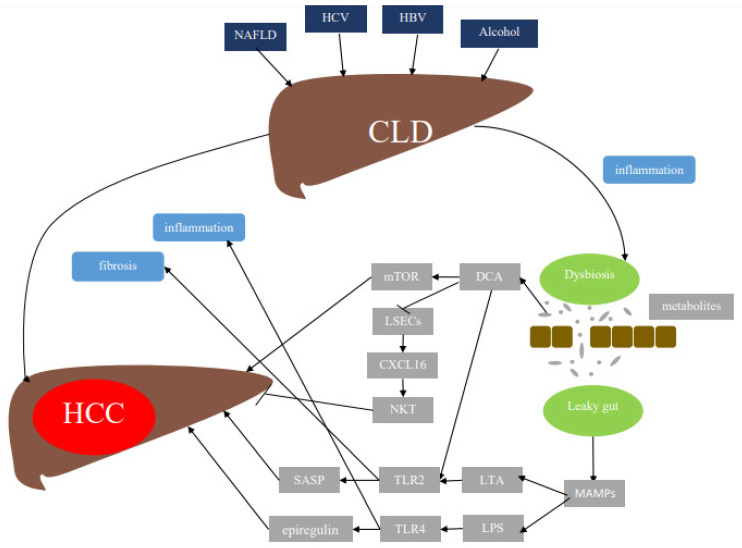
Mechanisms by which the gut microbiome plays a role in hepatocellular carcinoma. Besides the known risk factors for HCC, which include NAFLD, HBV, HCV, and alcohol, dysbiosis and leaky gut resulting from dysfunctional microbiota represent two other major factors leading to hepatic carcinogenesis. As such, several pathways are initiated, leading to HCC, which are detailed above. HCC: hepatocellular carcinoma; CLD: chronic liver disease; NAFLD: non-alcoholic fatty liver disease; HCV: hepatitis C virus; HBV: hepatitis B virus; MAMPs: microbiota-associated molecular patterns; TLR: toll-like receptor; LPS: lipopolysaccharide; LTA: lipoteichoic acid; SASP: senescence-associated secretory phenotype; DCA: deoxycholic acid; NKT: natural killer T cell; LSECs: liver sinusoidal cells; mTOR: mammalian target of rapamycin; CXCL-16: CXC motif ligand 16.

MAMPs include LPS, which is a cell wall component of gram-negative bacteria that triggers inflammation via TLR 4. TLR4 has been shown to mediate hepatic carcinogenesis via resident liver cells such as HSCs, macrophages, or hepatocytes. In addition to contributing to a chronic inflammatory state, TLR4 promotes the development of liver fibrosis and upregulates the expression of epiregulin, a potent HCC-promoting hepatomitogen, in HSCs [18]. Another MAMP is LTA and its receptor is TLR2. TLR2 is essential for the innate immune response to Gram-positive bacteria, being activated by bacterial lipoproteins and peptidoglycan. Once activated, TLR2 leads to a SASP, which seemed to be mediated in collaboration with DCA as well as Cox2 and prostaglandin E in HSCs [15]. In summary, the chronically injured liver is subject to increased exposure to a wide range of TLR ligands as well as other bacterial products and metabolites (Figure 1).

## 4. Changes in Gut Microbiota Associated with Different Liver Diseases

As the gut microbiome plays a critical role as an intermediary in the gut–liver axis, its composition and function evolve as changes in its host take place [19]. For instance, under normal physiological conditions, the majority of gut microbiota consists of microorganisms from *Firmicutes* phylum as well as from the *Actinobacteria* and *Verrucomicrobia* phyla [20]. Their role is to protect the host from overgrowth of pathogenic organisms. However, with different underlying chronic liver diseases (CLDs) come distinct changes in the gut microbiome profile, characterized mainly by loss of microbial diversity. The specific etiologies underlying CLD states have been characterized by unique microbial pathogens and loss of beneficial microorganisms, which are explicitly shown in Table 1.

Studies of the human gut microbiome and its association with CLDs have shown some heterogeneous results in terms of the type of abundant microorganisms constituting the gut microbiome of the same liver disease and in terms of abundance of specific bacteria (Table 1). For instance, *Bifidobacterium,* the gram-positive and non-spore forming bacilli, was decreased only in HBV patients and in patients with cirrhosis causing HCC. This bacterium belongs to the *Actinobacterium* phylum, which has beneficial effects on human health by acting as probiotic, thereby reducing plasma and intestinal endotoxin levels, changing gut microbiota contents, enhancing the gut–liver axis, and modulating the immunity [3,33]. On the other hand, the butyrate producing bacteria family *Ruminococcus* was less abundant in patients with HBV and HCC, but more abundant in patients with NAFLD and cirrhosis plus HCC. Another butyrate-producing bacteria family *Clostridia* was less abundant in patients with HBV, but more abundant in patients with cirrhosis. Butyrate, a kind of short chain fatty acid, is the major energy source of the intestinal mucosa and plays an important role in immunomodulation [34,35]. The phylum *Bacteriodetes* is composed of three large classes of gram-negative bacteria. Lower classifications of *Bacteroidetes* include *Prevotella*, *Bacteriodales*, *Flavobactericeae*, and so on. *Bacteriodetes* are abundant in patients with NAFLD and cirrhosis plus HCC, but decreased in patients with cirrhosis and HBV. Potentially pathogenic gram-negative bacteria family belonging to the *Proteobacteria* phylum such as *Enterobacteriaceae* comprises *Escherichia coli*, *Shigella*, *Proteus*, *Klebsiella,* and *Enterobacter*, which are increased in NAFLD, cirrhosis, HCC, and HBV. *Enterobacteriaceae* are ethanol-producing bacteria capable of causing liver damage and have been associated with levels of serum interleukin-6 (IL-6), IL-1, and tumor necrosis factor (TNF)-α [36,37]. *Firmicutes* phylum consists of gram-positive bacteria and includes both beneficial (*Clostridia, Clostridiaceae,* and *Ruminococcus*) and pathogenic (*Enterococcus* and *Streptococcus*) bacteria. *Streptococcus* are abundant in patients with cirrhosis and NAFLD, while *Enterococcus* are abundant in HCC. It is possible that the differences in microbial taxonomy are related to the causality of the disease, the geographical area, the target sequencing regions and depths of 16S ribosomal RNA gene, or the database that is selected.

## 5. Strategies to Manipulate Gut Microbiome for HCC Treatment/Prevention

Evidence suggests that the gut microbiota can promote the development of HCC through various mechanisms and may influence the efficacy of chemotherapy by modulating the host response to chemotherapeutic drugs, such as facilitation of drug efficacy, mediation of toxicity and abrogation of anticancer effects [38,39,40], the efficacy of immunotherapy by regulating the responses to the ICIs of patients with different cancers, and the efficacy of targeted therapy by modulating the metabolism and efficacy of some targeted drugs such as sorafenib and Wnt inhibitors [41,42,43]. Manipulation of the gut microbiota with probiotics, prebiotics, and FMT might be a novel, safe, and low-cost strategy to treat or prevent HCC.

### 5.1. Probiotics

Probiotics are a general term for active, beneficial microorganisms that colonize the human intestines and reproductive system. Probiotics have the potential to mitigate HCC risk by modulating host gut microbiota to promote growth of beneficial microbes and inhibit the growth of harmful ones [44]. Besides the traditional probiotic genera *Bifidobacterium* and *Lactobacillus*, a new group of probiotic bacteria, the so-called ‘next generation probiotics’, is currently emerging that mainly belong to butyrate-producing members of *Clostridium* clusters IV and XIVa (e.g., *Faecalibacterium prausnitzii*) or to the health-promoting mucin degraders *Akkermansia muciniphila*.

Probiotic bacteria can reduce the risk of HCC pathogenesis through multiple processes. For instance, probiotic bacteria promote the growth of beneficial gut microbes that produce anti-inflammatory metabolites with tumor suppression activity. Prohep, a novel probiotic mixture of *L. rhamnosus*, *E. coli* Nissle 1917, and heat inactivated VSL#3 (1:1:1), has been shown to shift the gut microbial community toward certain beneficial bacteria, including the *Prevotella* and *Oscillibacter*, which are known producers of anti-inflammatory metabolites, which subsequently reduced the Th17 polarization and promoted the differentiation of anti-inflammatory Treg/Tr1 cells in the gut [45]. Moreover, supplementation with probiotics attenuates HCC pathogenesis by downregulating the expression of TLR-induced inflammation. In Wistar rats with thioacetamide-induced liver cirrhosis, early administration of *L. plantarum* significantly decreased the expression of TLR4, CXCL9, and PREX-2 together with improvement in liver function [46].

Probiotic bacteria also have the ability to promote the epigenetic modulation of host gene expression to mitigate the pathogenesis of HCC. The probiotic bacteria *L. acidophilus* and *B. bifidum* reduced the expression of oncomirs (miR-155 and miR-221) and the oncogenes BCL2-like 2 (Bcl-w) and Kristen rat sarcoma viral oncogene homolog (KRAS) in the liver of mice treated with the colon carcinogen azoxymethane. Moreover, mice supplemented with these probiotics had an overexpression of the tumor suppressor miR-122 and tumor suppressor gene transcription factor PU.1 [47]. *L. paraplantarum* probiotic bacteria can reduce the diabetes-induced DNA damage in the livers of albino Wistar rats [48]. A novel probiotic mixture of *S. cerevisiae* and *L. acidophilus* enriched with selenium and glutathione synergistically prevented carbon tetrachloride (CCl4)-induced liver fibrosis by the activation of silent information regulator 1 (SIRT1) in hepatocytes. SIRT1 is a member of class III group of HDAC. Activation of SIRT1 can ameliorate the hepatic oxidative stress, ER stress, and inflammation induced by CCl4 in the rat livers, as indicated by reduced serum ALT and AST activities [49].

The antiviral activity of probiotics can be beneficial to mitigate HCC risk by preventing chronic HBV and HCV infections. Treatment HepG2 cells with extract of *B. adolescentis* resulted in a reduction of HBV viral load and cellular degeneration [50]. In HCV subjects, *E. faecalis* reduced the serum levels of liver damage markers ALT and AST, but failed to reduce HCV viral load [51]. Administration of probiotic bacteria increased the response rate to pegylated IFN-α and ribavirin treatment by 25% [52].

Moreover, probiotics prevent hepatic lipotoxicity by ameliorating obesity. In NAFLD patients, supplementation with the probiotic bacteria *L. acidophilus* and *B. lactis* can ameliorate liver damage, as indicated by reduced serum levels of ALT, AST, and total cholesterol [53]. In obese NAFLD patients, probiotic administration significantly reduced body weight and total body fat content. Moreover, probiotic administration decreased hepatic inflammation by downregulating the pro-inflammatory cytokine TNF-α [54,55].

Another method by which probiotics mitigate HCC pathogenesis is by controlling aflatoxin contamination. Supplementation with the yogurt containing the probiotic bacteria *S. thermophilus*, *L. rhamnosus*, and *W. cibaria* significantly reduced the urine availability of aflatoxin metabolites [56]. Finally, probiotic bacteria can biotransform non-nutritional dietary components such as proanthocyanidin into simpler metabolites with anticancer effects against HCC. For instance, biotransformed proanthocyanidins inhibit the proliferation of HepG2 cells by depleting mitochondria. The effective concentration of biotransformed proanthocyanidins is significantly low compared with the non-biotransformed material [57].

Overall, probiotics represent a new potential therapeutic strategy for HCC. Probiotic strains not only are a safe and less expensive therapeutic approach, but also can be tailored to different ages. Many more studies are required to clarify how to choose the specific probiotics for different sexes, ages, and diets.

### 5.2. Fecal Microbial Transplantation (FMT)

FMT is a new technique that transplants the functional flora from healthy human feces into the gastrointestinal tract of patients in order to reconstitute new beneficial intestinal flora [58]. FMT may repress the development of HCC by modulating the gut microbiome, reducing the production of some cytotoxic metabolites or inflammatory mediators and reversing the dysbiosis of the gut flora [59]. FMT is an effective treatment against recurrent *Clostridium difficile* infection. Moreover, it was shown to be a promising therapy for the management of several non-communicable disorders, including inflammatory bowel diseases and metabolic disorders [60]. FMT has increased in popularity because of its efficacy and ease of use and is being evaluated in clinical trials for NASH, NAFD, hepatitis, and cirrhosis. However, thus far, there have been few studies on the role of FMT in the treatment of HCC. More animal studies are required to prove the utility and safety of FMT. Possible drawback to its usage may include risk of disease transmission between the donor and recipient, patients’ acceptance, undesirable outcomes, and the uncertain impacts on the recipient’s immune system [59].

### 5.3. Prebiotics

Prebiotics are a dietary supplement that can selectively stimulate the growth and activity of bacteria and have a beneficial effect on the host [61]. Prebiotics can also restore the stability of the microbial community and reduce proinflammatory pathways that trigger hepatocarcinogenesis [62]. Among the most researched prebiotics, dietary polyphenols are of key importance. They include phenolic acids, flavonoids, and lignins found in nuts, wine, tea, fruits, and vegetables. Polyphenols, among other dietary substances such as coffee, vanadium, dietary fiber, fruits, and vegetables, show encouraging results in terms of chemoprevention in HCC [63]. Tea polyphenols possess potent antioxidant and anti-inflammatory properties and modulate several signaling pathways and provide an effective and promising alternative for the chemoprevention and treatment of HCC [64]. Moreover, curcumin, a major pigment of turmeric, is a natural antioxidant possessing a variety of pharmacological activities and therapeutic properties. Curcumin has shown anti-angiogenic properties in hepatocellular carcinoma cells (HepG2)-implanted nude mice [65] and induces apoptosis through mitochondrial hyperpolarization and mtDNA damage in HepG2 cells [66]. Moreover, curcumin effectively inhibits *N*-diethylnitrosamine (DEN)-induced murine hepatocarcinogenesis [67]. Resveratrol belongs to the stilbene group and is a main component of wine. Resveratrol inhibits urokinase-type plasminogen activator expression and the metastasis of HCC cells and is a powerful chemopreventative agent. The inhibitory effects were associated with the downregulation of the transcription factors of SP-1 signaling pathways [68]. Another prebiotic, the flavonoid quercetin, ameliorates nitric oxide production and nuclear factor NF-κB activation in IL-1β-activated rat hepatocytes [69].

## 6. Immunotherapy for HCC

Immune checkpoint blockade has become a turning point in the treatment of HCC, whereby it induces its antitumor effect by modulating the immune system [70]. Immune checkpoint inhibitors (ICIs), including programmed cell death protein-1 (PD-1) antibodies and programmed cell death 1 ligand 1 (PD-L1) antibodies, are potential therapeutic strategies for the treatment of HCC (Table 2).

Nivolumab is an anti-PD-1 antibody that was assessed primarily in the phase I/II nonrandomized CheckMate 040 trial [71]. The trial included a total of 262 patients; 48 patients in a dose-escalation phase and 214 patients in a dose-expansion phase. The overall response rate (ORR) was 20% and the disease control rate was 64% with Nivolumab 3 mg/kg in the dose-expansion phase compared with 15% and 58% in patients receiving the dose-escalation phase, respectively [71]. Further analysis from this trial revealed a median duration of response of 17 months in sorafenib-naïve patients and 19 months in patients treated previously with sorafenib. Moreover, the 18-month overall survival (OS) rates were 57% and 44%, respectively [72]. Based on these results, the FDA granted accelerated approval for nivolumab for patients with HCC who progressed on or after sorafenib. The Phase III CheckMate 459 trial compared nivolumab to sorafenib in the first-line treatment of advanced HCC. Median OS was 16.4 months for nivolumab and 14.7 months for sorafenib (HR 0.85 [95% CI: 0.72–1.02]; *p* = 0.0752). ORR was 15% for nivolumab (14 patients with complete response (CR)) and 7% for sorafenib (5 patients with CR) [73].

Pembrolizumab is another anti-PD-1 antibody that was assessed in the non-randomized, open-label phase II KEYNOTE-224 trial. The trial included 104 patients with advanced HCC that were intolerant to sorafenib or have progressed on it. An objective response was seen in 18 (17%; 95% CI 11–26) out of 104 patients. The best overall responses were 1 (1%) complete and 17 (16%) partial responses; meanwhile, 46 (44%) patients had stable disease, 34 (33%) had progressive disease, and six (6%) patients were not assessable [74]. Based on these results, the FDA granted accelerated approval for the use of pembrolizumab in patients progressing on sorafenib. Another phase III trial comparing pembrolizumab to placebo in the second-line treatment of advanced HCC did not meet its primary endpoints (OS and PFS) based on the rigorous statistical plan [75]. The combination of lenvantanib, an inhibitor of vascular endothelial growth factor receptor (VEGFR), of fibroblast growth factor receptor, of platelet-derived growth factor receptor (PDGFR), and other growth signaling kinases, and pembrolizumab was assessed in the phase Ib trial of 104 patients with unresectable HCC [76]. This combination is currently being investigated in a phase III trial against lenvantanib alone as a front-line therapy for unresectable or metastatic HCC (NCT03713593).

Atezolizumab is an anti-PD-L1 antibody that has been assessed mainly in combination with the VEGF inhibitor bevacizumab. This combination showed an ORR of 34% in patients with metastatic or unresectable HCC in a phase Ib trial [77]. Further analysis from the phase III IMbrave150 trial showed superior results of this combination over sorafenib in the first-line treatment of unresectable HCC [78]. However, prior to initiation of this regimen, patients should undergo endoscopic evaluation and management of esophageal varices within 6 months prior to treatment and based on the assessment of bleeding risk.

## 7. Influence of Gut Microbiome on Cancer Immunotherapy

A number of recent studies suggest that manipulating the microbiota may modulate the response to cancer immunotherapy. Oral administration of *Bifidobacterium* alone improved tumor control to the same degree as PD-L1 specific antibody therapy, and combination treatment nearly abolished tumor outgrowth. Augmented dendritic cell function leading to enhanced CD8^+^ T cell priming and accumulation in the tumor microenvironment mediated the effect in melanoma [80]. Another study examining oral and gut microbiota profiles in melanoma patients receiving PD-1 immunotherapy (n = 112) revealed significant differences in the diversity and composition of the patient gut microbiome of responders versus non-responders to immunotherapy. Analysis of 43 patient fecal microbiome samples by 16*S* ribosomal RNA gene sequencing showed an enrichment of *Clostridiales*, *Ruminococcaceae*, and *Faecalibacterium* in responders to anti-PD-1 treatment and *Bacteroidales* in non-responders. Twenty-five samples from the same cohort were analyzed by whole genome shotgun sequencing, confirming the enrichment of *Feacalibacterium* spp. in responders [81]. Analysis of baseline stool samples from metastatic melanoma patients before immunotherapy treatment, through an integration of 16*S* ribosomal RNA gene sequencing, metagenomic shotgun sequencing, and quantitative polymerase chain reaction for selected bacteria, revealed a significant association between commensal microbial composition and clinical response. Bacterial species more abundant in responders included *Bifidobacterium longum*, *Collinsella aerofaciens*, *Enterococcus faecium*, *Lactobacillus animalis*, *Parabacteroides merdae*, *Roseburia intestinalis*, *and Veillonella parvula* [82]. Moreover, germ-free mice that were colonized with bacteria were shown to be enriched in murine and human responders to ICIs, immune responsiveness was augmented via increased T helper 1 response, increased frequency of tumor-residing Batf3-lineage dendritic cells, and decreased frequency of colon-derived peripheral regulatory T-cells. Moreover, baseline gut microbiota enriched with *Faecalibacterium* and other *Firmicutes* was associated with beneficial clinical response to immune checkpoint inhibitor targeting cytotoxic T-lymphocyte-associated protein 4 (CTLA-4) (Ipilimumab) in melanoma patients [83]. In non-small cell lung cancer and renal cell carcinoma, the commensal that was most significantly associated with a favorable clinical outcome in both cancer types was *Akkermansia muciniphila* [84]. In pancreatic ductal adenocarcinoma, bacterial ablation was associated with immunogenic reprogramming of the PDA tumor microenvironment, including a reduction in myeloid-derived suppressor cells and an increase in M1 macrophage differentiation, promoting Th1 differentiation of CD4^+^ T cells and CD8^+^ T cell activation. Bacterial ablation also enabled efficacy for checkpoint-targeted immunotherapy by upregulating PD-1 expression [85]. These lines of evidence indicated that specific commensal microbes may shape patients’ responses to ICI immunotherapy, even though the gut bacteria that were associated with response across these published studies do not overlap.

Chronic antibiotic therapy is known to lead to gut dysbiosis and may disrupt this association, potentially diminishing the benefit of ICIs. Recently, several groups have reported a negative correlation between antibiotic exposure and outcomes for patients receiving treatment with ICIs for advanced solid cancers. In a retrospective study examining the influence of broad spectrum antibiotics on immunotherapy for advanced cancer, the use of antibiotics resulted in shorter progression free survival and OS [86]. The results from phase 1 trials in patients with renal cell carcinoma and non-small cell lung cancer showed that antibiotic use within 30 days of initiating ICI was associated with worse OS [87]. Antibiotic use in advanced non-squamous non-small cell lung cancer patients receiving ICI as second or later lines was identified as the only parameter statistically significantly associated with progression free survival and OS [88]. Moreover, in patients with advanced epithelial tumors, treatment with antibiotics inhibited the clinical benefit from ICIs [84]. Patients treated with antibiotics had significantly lower progression-free survival and OS rates compared with patients who had not received antibiotics. FMT from cancer patients who responded to ICIs into germ-free or antibiotic-treated mice ameliorated the antitumor effects of PD-1 blockade, whereas FMT from non-responding patients failed to do so. Metagenomics of patient stool samples at diagnosis revealed correlations between clinical responses to ICIs and the relative abundance of *Akkermansia muciniphila*. Oral supplementation with *A. muciniphila* after FMT with non-responder feces restored the efficacy of PD-1 blockade in an interleukin-12-dependent manner by increasing the recruitment of CCR9+CXCR3+CD4+ T lymphocytes into mouse tumor beds [84]. In a phase 1 clinical trial to assess the safety and feasibility of FMT and reinduction of anti-PD-1 immunotherapy in 10 patients with anti-PD-1-refractory metastatic melanoma, clinical responses were seen in three patients. Treatment with FMT was associated with favorable changes in immune cell infiltrates and gene expression profiles in both the gut lamina propria and the tumor microenvironment [89]>. Moreover, gut microbiota may secrete modulators or generate metabolites to improve HCC cells’ sensitivity to apoptosis induction and increase the response to ICI in advanced HCC patients [90].

## 8. Impact of Gut Microbiome on HCC Immunotherapy and Potential Use of Gut Microbiome Targeting Approaches

Zheng et al. reported the response to anti-PD-1 antibody immunotherapy in patients with HCC refractory to sorafenib [91]. Responders included those patients with complete response, partial response, or stable disease. Fecal samples were collected at intervals. In this study, non-responders had increased *Proteobacteria* from the third week, which became dominant by week twelve. However, responders had enriched *Akkermansia muciniphila* and *Ruminococcaceae* spp. [91]. These results suggest that the gut microbiome could possibly affect the outcome of anti-PD-1 immunotherapy in HCC patients.

Hepatic cirrhosis is often an underlying condition in HCC patients. Cirrhosis is associated with an extreme dysbiosis, which, in some circumstances, can contribute to drug resistance. It is thus reasonable to speculate that modulating the gut microbiome very likely has an impact on the treatment of HCC. Studies addressing molecular interactions underlying the effects of the microbiota on HCC development and antitumor immune responses are currently being pursued by different groups. For instance, a multicenter, randomized, double-blind, placebo-controlled study of nutritional supplementation with probiotics to prevent the development of HCC in cirrhosis patients (NCT03853928) will start recruiting patients. Another trial (NCT02021253) examined the effect of the administration of probiotics on intestinal barrier function in patients with chronic liver disease (fibrosis stage F3 or F4) operated on for HCC. A clinical trial combining vancomycin treatment with immune checkpoint blockade has recently opened at the National Cancer Institute (NCT03785210). This study will hopefully answer whether combining checkpoint inhibition with selective manipulation of the microbiota will be beneficial in patients with HCC.

## 9. Conclusions

Based on the growing body of evidence, it is becoming clear that modulation of the gut microbiome poses as a potential adjunct to current anti-cancer therapeutic strategies. Given that patients with HCC and other CLDs are subject to dysbiosis, it is enticing to speculate that dysbiosis is at the basis of immunotherapy failure in some patients and modulation of the gut microbiome in a way to overcome the state of dysbiosis may have a strong therapeutic effect in patients with HCC. For now, it is still not definite whether the current findings on the role of the gut microbiome in antitumor immune responses from animal models, as well as from patients with other tumor types, also apply to patients with HCC. New investigations on the gut microbiome, especially those focusing on fecal microbiota transplantation/probiotics, are clearly warranted to assist in the development of new paradigms and personalized treatments to enhance immunotherapy of HCC

## Figures and Tables

**Table 1:** Human studies involving gut microbial composition in various CLD states.

CLD Type	Microorganism	Reference
NAFLD	↓ *Prevotella* ↑ *Proteobacteria* ↑ *Fusobacteria* ↑ *Erysipelotrichaceae* ↑ *Enterobacteriaceae* ↑ *Lachnospiraceae* ↑ *Escherichia Shigella* ↑ *Streptococcaceae*	[21]
	↓ *Firmicutes* ↑ *Bacteroidetes*	[22]
	↓ *Prevotella* ↑ *Bacteroides* ↑ *Ruminococcus*	[23]
	*↑ Escherichia coli* *↑ Bacteroides vulgatus*	[24]
Cirrhosis	↑ *Enterobacteriaceae* ↑ *Enterococcus*	[25]
	↓ *Bacteroidetes* ↑ *Proteobacteria* ↑ *Fusobacteria* ↑ *Enterobacteriaceae* ↑ *Streptococcaceae* ↑ *Veillonellaceae*	[26]
	↓ *Bacteroides* ↑ *Prevotella* ↑ *Clostridium* ↑ *Streptococcus* ↑ *Veillonella*	[27]
	↓ *Akkermansia* ↑ *Enterobacteriaceae* ↑ *Streptococcaceae*	[28]
HBV	↓ *Bifidobacterium* ↓ *Clostridiaceae* ↓ *Clostridia* ↓ *Ruminococcus* ↑ *Klebsiella* ↑ *Escherichia coli* ↑ *Proteus* ↑ *Enterobacter*	[29]
	↓ *Bacteroidetes* ↑ *Proteobacteria*	[30]
Cirrhosis + HCC	↓ *Bifidobacterium* ↑ *Bacteroidetes* ↑ *Ruminococcaceae*	[28]
HBV + HCC	↓ *Verrucomicrobia* ↑ *Actinobacteria*	[31]
HCC	↓ *Faecalibacterium* ↓ *Ruminococcus* ↓ *Ruminoclostridium* ↑ *Escherichia-Shigella* ↑ *Enterococcus*	[32]

↑: increased; ↓: decreased; NAFLD: non-alcoholic fatty liver disease; HBV: hepatitis B virus; HCC: hepatocellular carcinoma.

**Table 2:** Trials involving immune checkpoint inhibitors for the treatment of HCC.

Treatment	Patients	Clinical Phase	PFS (Months, 95% CI)	Median OS (Months, 95% CI)	RR (%, 95% CI)	Reference
Nivolumab	Advanced HCC	Phase I/II	3.4 (1.6–6.9), for DS 4.1 (3.7–5.5), for EX	15.0 (9.6–20.2), for DS NR, for EX	15% (6–28), for DS 20% (15–26), for EX	[71]
Nivolumab	Advanced HCC	Phase III	3.7 (3.1–3.9)	16.4 (13.9–18.4)	15%	[73]
Sorafenib			3.8 (3.7–4.5)	14.7 (11.9–17.2) (HR 0.84, *p* = 0.0419)	7%	
Nivolumab plus Ipilumab	Advanced HCC	Phase I/II		22.8 (95% CI, 9.4 NR)	32% (95% CI, 20–47%)	[79]
Pembrolizumab	Advanced HCC	Phase II	4.8 (3.4–6.6)	12.9 (9.7–15.5)	17% (11–26)	[74]
Pembrolizumab	Second-line, Advanced HCC	Phase III	3.0 (2.8–4.1)	13.9 (11.6–16.0)	18.3 (14.0–23.4)	[75]
Placebo			2.8 (2.5–4.1)	10.6 (8.3–13.5) (HR 0.781, *p* = 0.023)	4.4 (1.6–9.4)	
Pembrolizumab plus Lenvatinib	Unresectable HCC	Phase Ib	9.3	22.0	46.0% (36.0–56.3)	[76]
Atezolizumab plus bevacizumab	Unresectable HCC	Phase 1b			34%	[77]
Atezolizumab plus Bevacizumab	Unresectable HCC	Phase III	6.8 (5.7–8.3)	67.2% (61.3–73.1)		[78]
Sorafenib			4.3 (4.0–5.6) (HR 0.59, *p* < 0.001)	54.6% (45.2–64.0) 12 months response		

DS: dose-escalation group; EX: dose-expansion group; NR: not reached; HR: hazard ratio

## Abbreviations

HCC	Hepatocellular carcinoma
LPS	Lipopolysaccharides
MAMPs	Microbiota-associated molecular patterns
DCA	Deoxycholic acid
LSEC	Liver sinusoidal cells
NKT	Natural killer T cell
TLR2	Toll-like receptor 2
HSC	Hepatic stellate cells
LTA	Lipoteichoic acid
SASP	Senescence-associated secretory phenotype
mTOR	Mammalian target of rapamycin
CLD	Chronic liver disease
NAFLD	Non-alcoholic fatty liver disease
HCV	Hepatitis C virus
HBV	Hepatitis B virus
IL-6	Interleukin-6 (IL-6)
TNF-α	Tumor necrosis factor-α
ICIs	Immune checkpoint inhibitors
PD-1	Programmed cell death protein-1
PD-L1	Programmed cell death 1 ligand 1
ORR	Overall response rate
OS	Overall survival
CR	Complete response
VEGFR	Vascular endothelial growth factor receptor
PDGFR	Platelet-derived growth factor receptor
CTLA-4	Cytotoxic T-lymphocyte-associated protein 4
FMT	Fecal microbiota transplantation
SIRT1	Silent information regulator 1
CCL4	Carbon tetrachloride
HepG2	Hepatocellular carcinoma cells
DEN	*N*-diethylnitrosamine
